# Competing Activities of Heterotrimeric G Proteins in *Drosophila* Wing Maturation

**DOI:** 10.1371/journal.pone.0012331

**Published:** 2010-08-23

**Authors:** Natalya Katanayeva, Damir Kopein, Reto Portmann, Daniel Hess, Vladimir L. Katanaev

**Affiliations:** 1 Department of Biology, University of Konstanz, Konstanz, Germany; 2 Friedrich Miescher Institute for Biomedical Research, Basel, Switzerland; 3 Institute of Protein Research, Russian Academy of Science, Pushchino, Russia; University of Dayton, United States of America

## Abstract

*Drosophila* genome encodes six alpha-subunits of heterotrimeric G proteins. The Gαs alpha-subunit is involved in the post-eclosion wing maturation, which consists of the epithelial-mesenchymal transition and cell death, accompanied by unfolding of the pupal wing into the firm adult flight organ. Here we show that another alpha-subunit Gαo can specifically antagonize the Gαs activities by competing for the Gβ13F/Gγ1 subunits of the heterotrimeric Gs protein complex. Loss of Gβ13F, Gγ1, or Gαs, but not any other G protein subunit, results in prevention of post-eclosion cell death and failure of the wing expansion. However, cell death prevention alone is not sufficient to induce the expansion defect, suggesting that the failure of epithelial-mesenchymal transition is key to the folded wing phenotypes. Overactivation of Gαs with cholera toxin mimics expression of constitutively activated Gαs and promotes wing blistering due to precocious cell death. In contrast, co-overexpression of Gβ13F and Gγ1 does not produce wing blistering, revealing the passive role of the Gβγ in the Gαs-mediated activation of apoptosis, but hinting at the possible function of Gβγ in the epithelial-mesenchymal transition. Our results provide a comprehensive functional analysis of the heterotrimeric G protein proteome in the late stages of *Drosophila* wing development.

## Introduction

G protein-coupled receptors (GPCRs) represent the most populous receptor family in metazoans. Approximately 380 non-olfactory GPCRs are encoded by the human genome [1], corroborated by ca. 250 GPCRs in insect genomes [2], [3], making 1–1.5% of the total gene number dedicated to this receptor superfamily in invertebrates and mammals. GPCRs transmit their signals by activating heterotrimeric G protein complexes inside the cell. A heterotrimeric G protein consists of a GDP-bound α-subunit and a βγ-heterodimer. Ligand-stimulated GPCR serves as a guanine nucleotide-exchange factor, activating the GDP-to-GTP exchange on the Gα-subunit. This leads to dissociation of the heterotrimeric complex into Gα-GTP and βγ, which transmit the signal further inside the cell [4].

The β- and γ-subunit repertoire of the *Drosophila* genome is reduced as compared with that of mammals: only two Gγ and three Gβ genes are present in flies (Table 1). Gγ30A and Gβ76C are components of the fly phototransduction cascade and are mostly expressed in the visual system [5], [6]. Gγ1 and Gβ13F have been implicated in the asymmetric cell divisions and gastrulation [7], [8], while the function of Gβ5 is as yet unknown.

**Table 1:** The list of *Drosophila* Gα, Gβ, and Gγ subunits, with the information on their function and human homologies.

G protein subunit	synonyms	number of isoforms	sub-group	human ortholog (% identity)	described function
Gα subunits					
Gαo	G protein oα 47A, brokenheart (bkh)	2	Gαo/i	Gαo (82%)	Frizzled receptor signal transduction in the Wnt and planar cell polarity pathways [15]; control of asymmetric cell divisions in the sensory organ lineage [17]; feeding behavior [42]; learning and memory [43]; heart development [44], [45]; axonal growth/guidance [44]; blood-brain barrier formation [46]
Gαi	G protein αi subunit 65A	1	Gαo/i	Gαi1 (77%)	control of the asymmetric cell divisions in the neuroblast and sensory organ lineages [7]; blood-brain barrier formation [46]; Hedgehog signal transduction [47]
Gαs	G protein sα 60A	2	Gαs	Gαs (72%)	larval growth [19]; establishment of the neuro-muscular synapse [48]; post-eclosion wing maturation [22]
Gαf	G protein α 73B	1	Gαf	none (40% to Gαs)	none described
Gαq	G protein α49B	≥2	Gαq/11	Gαq (77%)	phototransduction [18]; olfaction [31]
concertina	cta	1	Gα12/13	Gα13? (55%)	gastrulation [13]
Gβ subunits					
Gβ13F		1		Gβ1 (83%)	control of the asymmetric cell divisions in the neuroblast and sensory organ lineages [7]; gastrulation [7]; heart development [45]
Gβ76C	Gβe	1		none? (43% to Gβ1)	phototransduction [5]
Gβ5		1		Gβ5 (68%)	none described
Gγ subunits					
Gγ1		1		Gγ12? (44%)	control of the asymmetric cell divisions in the neuroblast and sensory organ lineages [8]; gastrulation [8]; heart formation [49]
Gγ30A	Gγe	1		Gγ13? (41%)	phototransduction [6]

Despite the fact that βγ can activate signal effectors [9], the main selectivity in GPCR coupling and effector activation is provided by the Gα-subunits [10]. Sixteen genes for the α-subunits are present in the human genome, and six in *Drosophila*. All human Gα-subunit subgroups are represented in *Drosophila* (Table 1): Gαi and Gαo belonging to the Gαi/o subgroup; Gαq belonging to the Gαq/11 subgroup; Gαs belonging to the Gαs subgroup, and concertina (*cta*) belonging to the Gα12/13 subgroup [10]. Additionally, *Drosophila* genome encodes for Gαf which probably represents an insect-specific subfamily of Gα-subunits [11].

Multiple functions have been allocated to different heterotrimeric G proteins in humans and flies [12], see Table 1. For example, in *Drosophila* development *cta* is a crucial gastrulation regulator [13], Gαo is important for the transduction of the Wnt/Frizzled signaling cascade [14], [15], and Gαi controls asymmetric cell divisions during generation of the central and peripheral nervous system [7] (the later in cooperation with Gαo [16], [17]). Gαq is the *Drosophila* phototransduction Gα-subunit, but probably has additional functions [18]. Pleotropic effects arise from defects in Gαs function [19], while the function of Gαf has not yet been characterized.

Among the developmental processes ascribed to the control by Gαs are the latest stages of *Drosophila* wing development. Newly hatched flies have soft and folded wings, which during the 1–2 hours post-eclosion expand and harden through intensive synthesis of components of the extracellular matrix. These processes are accompanied by epithelial-mesenchymal transition and apoptosis of the wing epithelial cells, producing a strong but mostly dead adult wing structure [20], [21], [22]. Expression of the constitutively active form of Gαs leads to precocious cell death in the wing epidermis, which results in failure of the closure of the dorsal and ventral wing sheets and accumulation of the hemolymph inside the wing, producing wing blistering [22], [23]. Conversely, clonal elimination of Gαs leads to autonomous prevention of the cell death. Kimura and co-workers have performed an extensive analysis of the signaling pathway controlling apoptosis at late stages of wing development [22]. They provide evidence suggesting that the hormone bursicon, synthesized in the head of post-eclosion *Drosophila* and secreted in the hemolymph, activates a GPCR *rickets* on wing epithelial cells, which signals through Gαs to activate the cAMP-PKA pathway, culminating at the induction of apoptosis [22]. However, the identity and importance of the βγ subunits in bursicon signaling, as well as possible involvement of other Gα proteins remained outside of their investigation. There also remain some uncertainties as to the phenotypic consequences of elimination of the bursicon-Gαs-PKA pathway in wings [21], [22], [24].

Here we describe a comprehensive functional analysis of the *Drosophila* heterotrimeric G protein proteome using loss-of-function and overexpression experiments. We show that loss of Gαs but not any other Gα-subunit leads to the failure of wing expansion after fly hatching. We also show that Gαo, but not another Gα, can compete with Gαs and thus antagonize its function. Finally, we identify the Gβ13F and Gγ1 as the βγ subunits of the heterotrimeric Gs complex responding to the epithelial-mesenchymal transition and cell death-promoting signal.

## Results

### Gαo, but not other Gα-subunits, in its GDP-loaded state prevented post-eclosion wing unfolding in Drosophila

**Figure 1:**
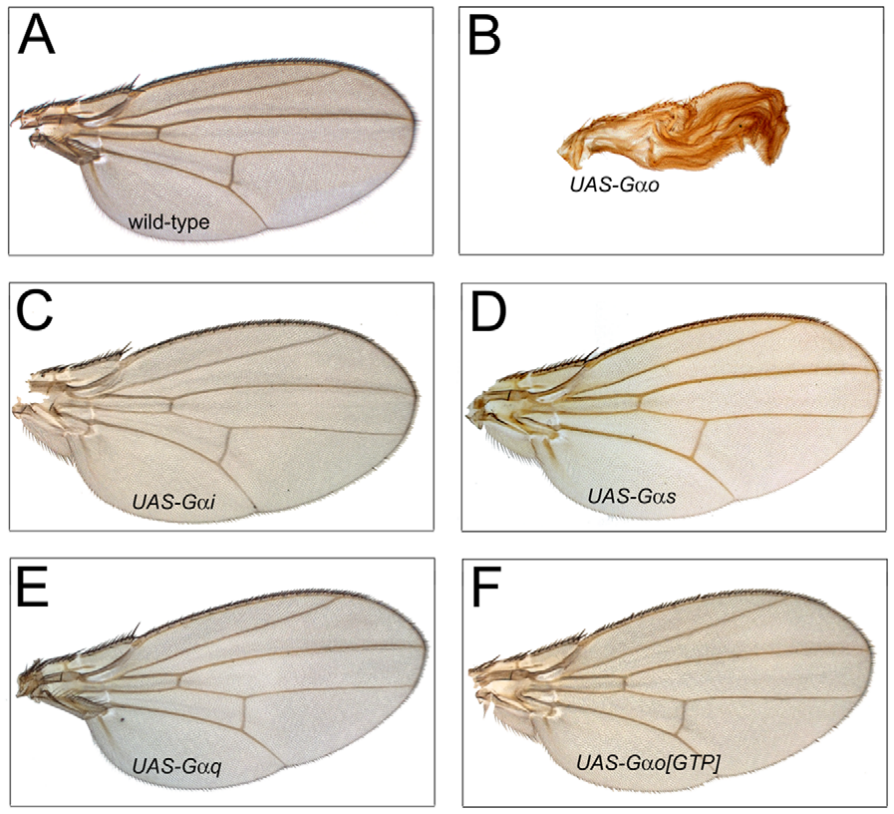
Overexpression of Gαo, but not other Gα-subunits, leads to the failure of wing expansion. Representative wings overexpressing Gαo (B), its activated form (Gαo[GTP], F), Gαi (C), Gαs (D), or Gαq (E) are shown along with the *MS1096-Gal4* driver line alone (A).

In the course of our studies of the role of the Gαo subunit of heterotrimeric G proteins in the Wnt and PCP signaling in *Drosophila* wing development [15] we came across an observation that overexpression of Gαo in *Drosophila* wings often led to the failure of wing expansion after fly hatching from the pupal case. Using the X-chromosome-located *MS1096-Gal4* driver line, we found that ca. 80% of the aged adult female flies and 90% of male flies had folded wings characteristic of the freshly eclosed flies - a phenomenon never observed with wild-type animals (Fig. 1A, B, Table 2). *MS1096-Gal4* drives strong expression in the dorsal domain and weaker expression in the ventral domain of the developing larval and pupal wing [25], [26], [27].

**Table 2:** Rescue of the wing expansion defect of *MS1096-Gal4, UAS-Gαo* flies by Gβγ or Gαs.

genotype	% folded wings, females	*n*, total wings analyzed, females	% folded wings, males	*n*, total wings analyzed, males
*UAS-Gαo*	79%	180	93%	41
*UAS-Gαo; UAS-Gβ13F; UAS-Gγ1*	3%	35	32%	22
*UAS-Gαo; UAS-Gαs*	18%	50	50% (ns)	6 (ns)
*UAS-Gαo; UAS-Gαs[GTP]*	50%	30	100% (ns)	9 (ns)

ns- the number of flies available for analysis is not significant. Note that the viability of the male flies expressing Gαo under the control of the X-linked *MS1096-Gal4* driver is reduced.

A similar overexpression of other Gα-subunits, Gαs, Gαi, or Gαq, did not produce this effect (Fig. 1C–E), suggesting that Gαo was unique in its ability to prevent wing expansion post-eclosion. Interestingly, the activated Q205L mutant form of Gαo, which stays constantly bound to GTP [15], [16], could not induce the folded wing phenotype (Fig. 1F). These data suggest that the GDP-, but not the GTP-loaded, form of Gαo upon overexpression binds and sequesters a specific protein required for the proper post-eclosion wing development.

### Proteomic analysis identifies very few proteins discriminatively interacting with Gαo in its GDP vs GTP form

**Figure 2:**
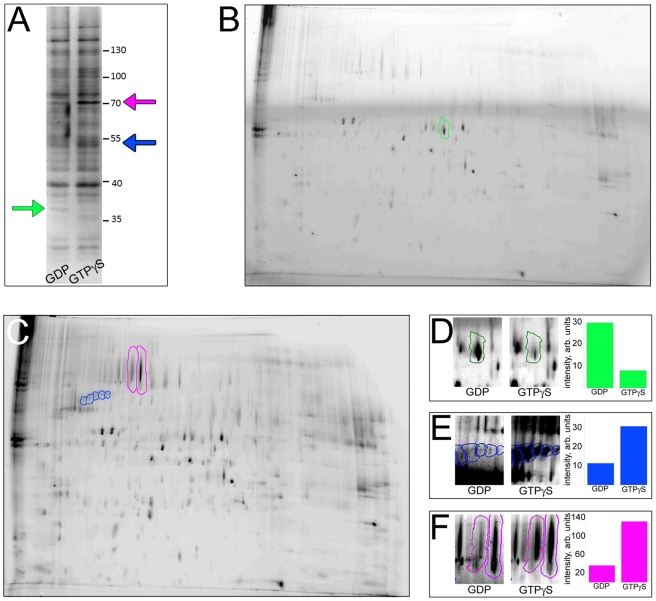
Proteomic analysis identifies a few *Drosophila* proteins specifically interacting with the GDP- or the GTP-loaded forms of Gαo. **A**. SDS-PAGE of the *Drosophila* head proteins retained by the GDP- or the GTPγS-loaded CNBr-immobilized Gαo. The single band enriched in the GDP-lane is indicated by the green arrow. The two bands enriched in the GTPγS-lane are indicated by the blue and magenta arrows. Positions of molecular weight markers are shown to the right of the gel. **B–C**. 2D-gel of the same samples as shown in (A), DIGE-labeled and loaded on the same gel, visualized in the Cy3-channel (B, Gαo-GDP-interacting proteins) and in the Cy5-channel (C, Gαo-GTPγS-interacting proteins). The spots enriched in one or the other samples are outlined in green, blue and magenta. **D–F**. High magnification of the spots enriched in the Gαo-GDP- *vs* the Gαo- GTPγS -interacting proteins, together with the quantification of the normalized intensity of these spots between the two samples. Quantification in (E, F) is presented as the sum of intensities of all the spots outlined (five in (E), two in (F)). The spot in (D) was identified as Gβ13F, spots 1 and 4 (from left to right) in (E) were identified as β1-Tubulin, spot 2 in (F) was identified as Hsc70-3; other spots failed to be identified by LC-MSMS.

In order to identify the protein(s) which might be sequestered by the overexpression of the wild-type (mostly GDP-loaded), but not the GTP-loaded form of Gαo during post-eclosion wing expansion, we performed a proteomic analysis of Gαo-binding partners which would bind specifically to its GDP- or its GTP-loaded states, but not to both forms. To this end, we bacterially expressed wild-type Gαo and immobilized it on CNBr-sepharose. These procedures resulted in Gαo which was approximately 50% active as determined in the GTP-binding assays [16]. The matrix was then preloaded with GDP or GTPγS (a non-hydrolysable GTP analog) and used to apply *Drosophila* head extracts. After washing, proteins retained were eluted with Urea and resolved on SDS-PAGE (Fig. 2A). We could identify three bands which bound preferentially to either nucleotide form of Gαo: two in the GTPγS-matrix (ca. 53 kDa and 71 kDa), and one in the GDP-matrix (ca. 37 kDa). These findings could be confirmed by high resolution protein separation using 2D-PAGE with DIGE labeling [28]. The three proteins from *Drosophila* head extracts discriminatively bound to either nucleotide form of Gαo in our experiment (Fig. 2B–F), suggesting that the majority of Gαo target proteins interact equally well with GDP- and GTP-loaded Gαo. The 53 kDa and 71 kDa proteins migrated as several spots on 2D-gels (Fig. 2C, E, F), which might indicate post-translational modifications of the proteins.

LC-MSMS after trypsin in-gel digestion was used to identify these three proteins. The 71 kDa protein was found to be the Heat-shock 70 kDa protein cognate 3 (gene name: Hsc70-3), the 53 kDa protein was identified as Tubulin β1-chain (gene name: β-Tubulin at 56D), and the 37 kDa protein exclusively binding to Gαo-GDP - as the Guanine nucleotide-binding protein subunit β-1 (gene name: Gβ13F subunit). While tubulins have previously been found to physically bind Gα-subunits [29], [30], binding of Hsc70-3 to a G protein has not been reported before. As for the Gβ13F subunit, the interaction of GDP-loaded Gαo with the βγ heterodimers is expected. However, we initially did not suspect that sequestration of βγ by overexpressed Gαo could be the reason for the wing unfolding defects, as other Gα-subunits would also be expected to sequester βγ, and yet were ineffective in preventing wing unfolding (Fig. 1).

### Gβ13F and Gγ1, but not other Gβ/γ subunits, are required for the post-eclosion wing unfolding

To test whether the post-eclosion Gαo-overexpression phenotype was due to sequestration of Gβγ, we first aimed at rescuing the Gαo phenotype by providing more βγ. To this end, we co-expressed Gαo, Gβ13F, and Gγ1 by the *MS1096-Gal4* driver line. Indeed, we found an overwhelming rescue of the wing expansion defect if Gβ13F/Gγ1 were co-overexpressed: only 3% of aged female wings and 32% of the male wings now remained folded, as compared to 79% and 93% of female and male flies, respectively, overexpressing Gαo alone (Table 2).

**Figure 3:**
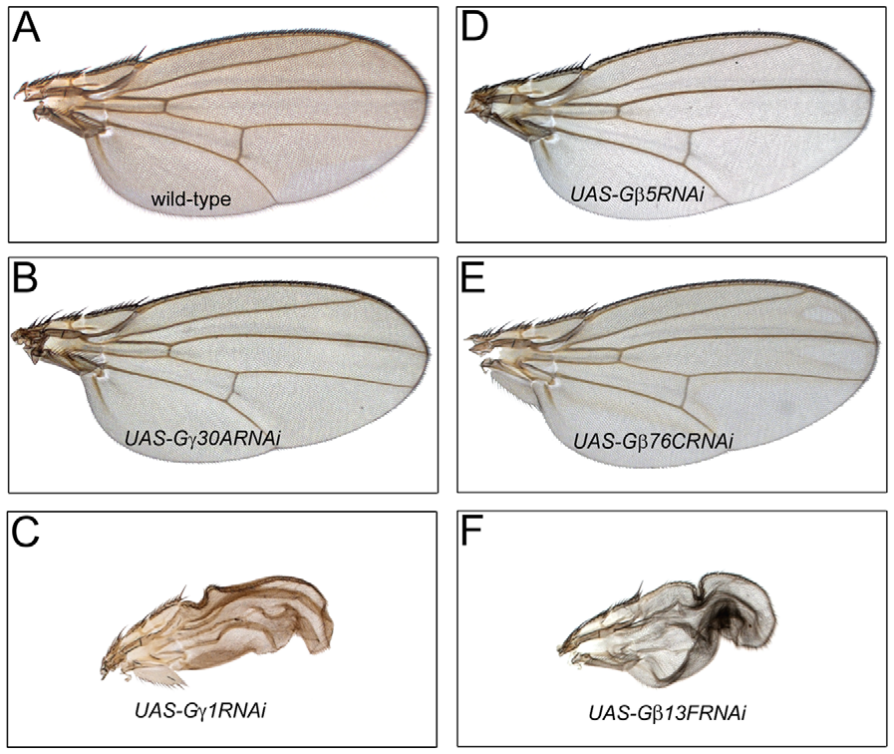
Downregulation of Gγ1 or Gβ13F, but not of any other Gβ or Gγ subunit, leads to the failure of wing expansion. Representative wings expressing the RNAi constructs targeting Gγ30A (B), Gγ1 (C), Gβ5 (D), Gβ76C (E), or Gβ13F (F) are shown along with the *MS1096-Gal4* driver line alone (A).

Next, to address the question whether Gβγ heterodimers were necessary for the post-eclosion wing development, we expressed RNAi lines targeting Gβ13F, Gβ5, Gβ76C, Gγ1, or Gγ30A by *MS1096-Gal4*. As shown on Fig. 3A-C, RNAi against Gγ1, but not Gγ30A, prevented wing expansion similarly to that induced by Gαo overexpression (Fig. 1B). When RNAi lines targeting the three Gβ-subunits were expressed, RNAi against Gβ13F, but not Gβ5 or Gβ76C, was found to prevent wing expansion (Fig. 3D–F). Flies homozygous mutant for the Gβ76C gene also showed no defects in wing development (data not shown). Other phenotypes of the downregulation of Gβ13F and Gγ1 suggested the role of this Gβγ heterodimer in the process of asymmetric cell divisions [17], Wnt signaling [14], and planar cell polarity (not shown). Altogether, our results point to a simple model in which overexpression of the wild-type Gαo, but not Gαi, Gαs, or Gαq, sequestered Gβ13F/Gγ1 required for the post-eclosion wing expansion in *Drosophila*.

### Gβ13F/Gγ1 constitute with Gαs the heterotrimeric G protein complex required for the post-eclosion wing expansion

**Figure 4:**
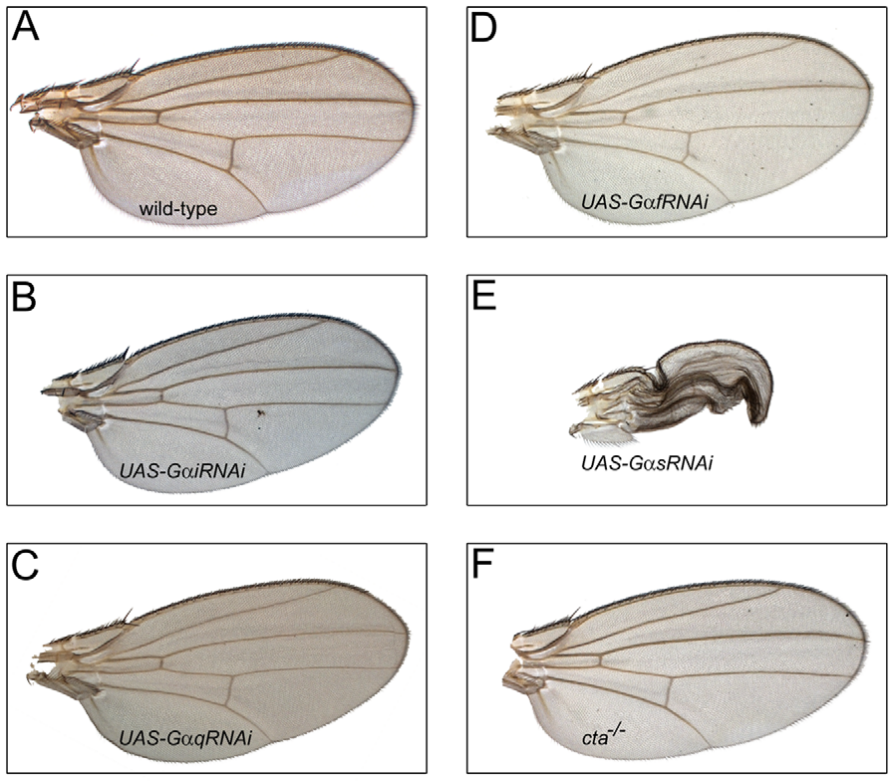
Downregulation of Gαs, but not other Gα-subunits, leads to the failure of wing expansion. Representative wings expressing the RNAi constructs targeting Gαi (B), Gαq (C), Gαf (D), or Gαs (E) are shown along with the *MS1096-Gal4* driver line alone (A) and the wing of the concertina homozygous mutant fly (*cta^−/−^*, F).

We supposed that Gαo competed for Gβ13F/Gγ1 with another Gα-subunit, thus inactivating a heterotrimeric G protein complex required for the proper wing expansion. To investigate the nature of this Gα subunit outcompeted by Gαo, we systematically removed all other Gα proteins by using loss-of-function mutations or targeted RNAi expression. RNAi-targeted downregulation was employed to target Gαi, Gαq, Gαf, and Gαs (Fig. 4A–F); of these constructs, those targeting Gαq and Gαi were previously shown efficient in downregulating target gene expression [16], [31]. Concertina was removed using the null allele [13]. Similar elimination of Gαo is not possible due to the requirement of this G protein for cell viability in the wing [15]. Gαo can be specifically uncoupled from GPCRs using the expression of pertussis toxin [29]; such whole wing expression of pertussis toxin does not result in any visible defects in wing expansion [16].

Out of all Gα tested, elimination of Gαs from the wing produced the wing unfolding defect similar to that induced by overexpression of Gαo or downregulation of Gβ13F/Gγ1 (Fig. 4E). In contrast, elimination of other Gα proteins in the wings did not produce visible defects (Fig. 4). Thus, we concluded that among different Gα-subunits only elimination of Gαs led to the wing unfolding defect. In agreement with this, we found that co-overexpression of Gαs together with Gαo strongly suppressed the ability of the latter to produce the folded wing phenotype (Table 2). Thus, the heterotrimeric G protein complex, consisting of the Gαs, Gβ13F, and Gγ1 subunits is required for the proper signaling regulating wing expansion post-eclosion, and can be antagonized by Gαo.

### The wing expansion defect is associated with, but is not caused by, prevention of cell death

**Figure 5:**
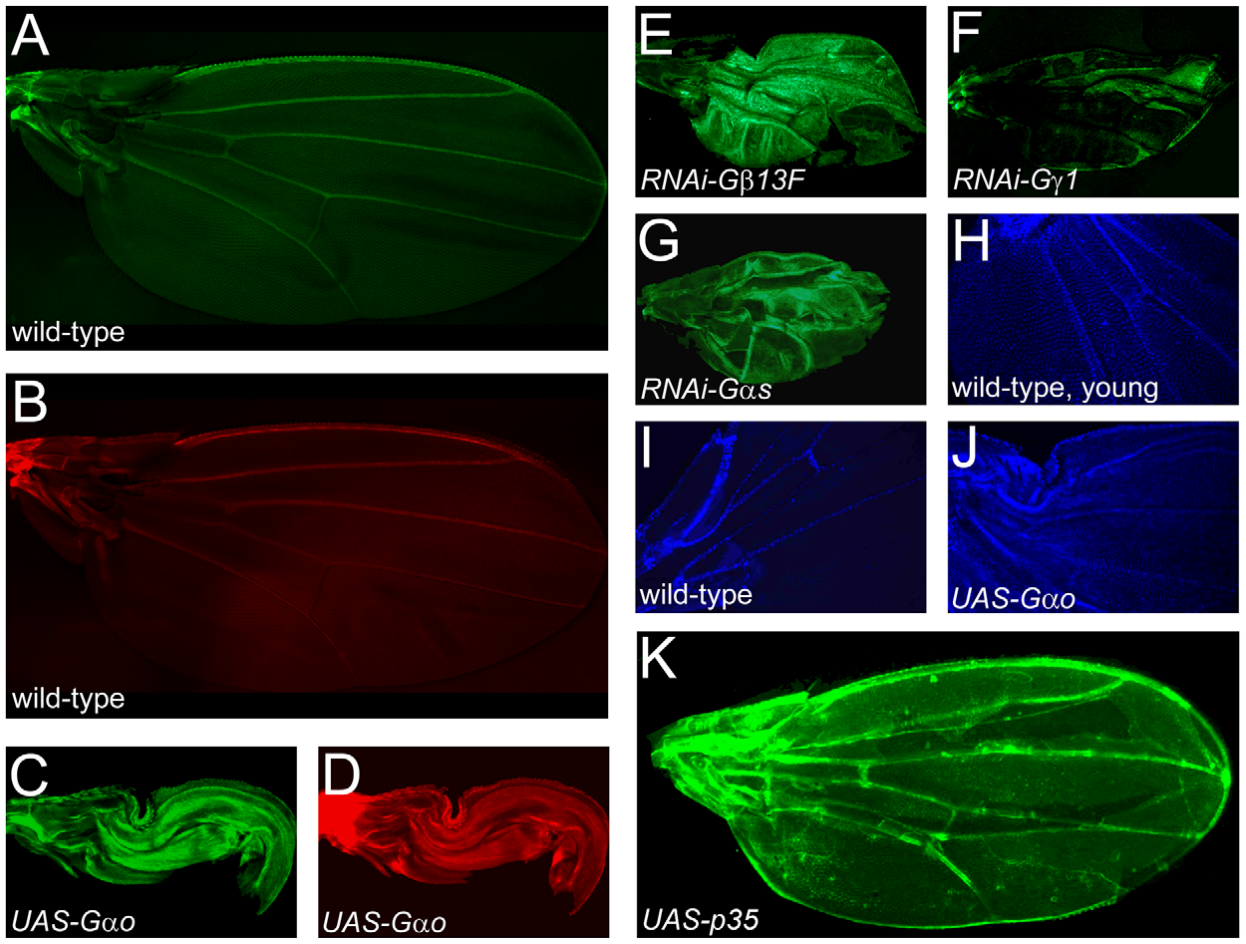
Prevention of apoptosis is associated with, but is not sufficient to induce, the failure of wing expansion. **A–B**. Wild-type wings are fully expanded and show GFP (A) or F-actin (B) staining only on the margin and along the veins, demonstrating that the adult wings are mostly dead structures. **C–G**. Downregulation of the Gs pathway by overexpression of Gαo (C, D) or by expression of RNAi constructs targeting Gβ13F (E), Gγ1 (F), or Gαs (G) leads to both failure of wing expansion and prevention of apoptosis, as visualized by persistence of F-actin- (D) and GFP-positive cells (C, E–G). **H–J**. DAPI nuclear staining. Overexpression of Gαo in aged wings leads to the DAPI staining pattern (J) characteristic of the young (ca. 1h-old, H) wild-type wings; aged wild-type wing only shows DAPI staining along the veins (I). **K**. Expression of the apoptosis inhibitor p35 prevents cell death throughout the wing as seen by persistence of GFP-positive cells, but does not cause the failure of wing expansion. All wings presented here are from *MS1096-Gal4; UAS-GFP* flies which are ≥1 day-old (except for the wing of panel (H)).

Clonal elimination of Gαs results in failure of the cell death in the wing [22]. Indeed, while aged flies retained live GFP- and rhodamine phalloidin-stained cells only along the veins and wing margin (Fig. 5A, B), we found that the *MS1096-Gal4*-driven expression of Gαo or RNAi constructs targeting Gβ13F, Gγ1, or Gαs all similarly resulted in maintenance of live cells within the wing blade of well-aged flies (Fig. 5C–G). To better resolve the remaining live cells, we performed the nuclear staining with DAPI [22], [24]. Young (ca. 1h-old) wild-type wings contain many DAPI-positive living cells (Fig. 5H), but aged wild-type wings showed DAPI staining only alone the veins (Fig. 5I). In contrast, wings of the Gαo-overexpressing flies up to six days old were still filled with DAPI-positive living cells (Fig. 5J). These data clearly show that the wing expansion failure is associated with the failure of cell death. However, is prevention of the cell death sufficient to cause the folded wing phenotype? To investigate this possibility, we expressed the baculovirus apoptosis inhibitor p35 in the entire wing under the *MS1096-Gal4* control. While apoptosis was efficiently prevented, wing expansion was normal in these wings (Fig. 5K). This data agrees with the similar observations obtained when p35 was expressed using other *Gal4* drivers [21], [22]. Cumulatively, our data suggest that apoptosis, being an important process during post-eclosion wing maturation, is unlikely to be the sole driving force behind wing expansion. Wing expansion seems more dependent on the epithelial-mesenchymal transition [21], [24], or perhaps requires both processes to act in concert. Elimination of the components of the heterotrimeric Gs proteins apparently leads to both the failure of epithelial-mesenchymal transition and apoptosis, leading cumulatively to the wing expansion defect.

### Overactivation of Gαs by cholera toxin mimics expression of the constitutively active mutant form of Gαs, not reproduced by overexpression of Gβ13F/Gγ1

**Figure 6:**
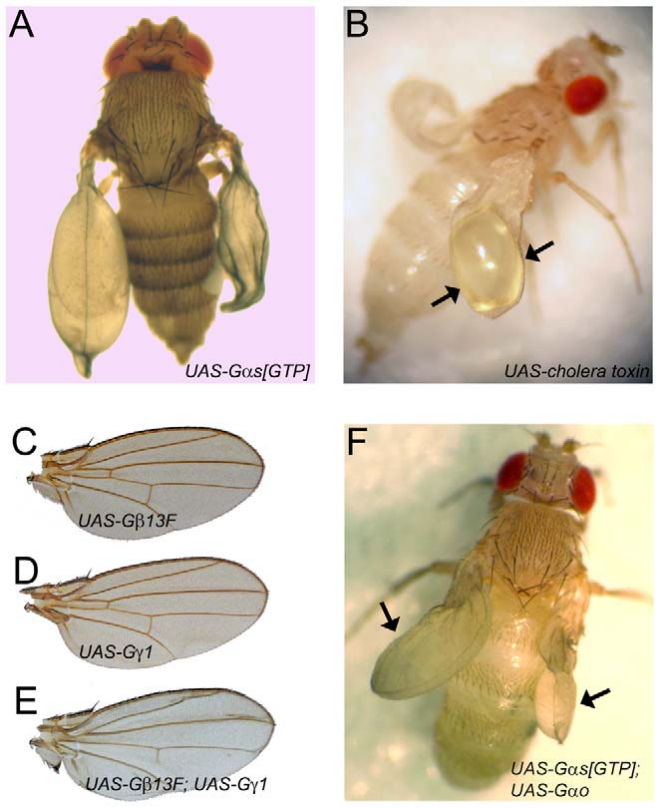
Overactivation of Gαs, but not Gβγ, leads to wing blistering due to precocious apoptosis. **A**. Expression of the constitutively active form of Gαs by multiple *Gal4* drivers produces the characteristic wing blistering. The picture shown represents an *OK10-Gal4/UAS-Gαs[GTP]* fly. **B**. Activation of the endogenous Gαs by expression of cholera toxin with multiple drivers also produces wing blistering (arrows). The picture shown represents a *Vg-Gal4, UAS-flp; UAS>w^+^>cholera toxin* fly. **C–E**. Expression of Gβ13F alone (C), Gγ1 alone (D), or both (E) by multiple drivers including *OK10-Gal4* never produces the wing blistering. The pictures shown represent wings of *MS1096-Gal4; UAS-Gβ/γ* flies. **F**. Sequestration of Gβγ by Gαo does not prevent wing blistering induced by the constitutively active form of Gαs. The picture shown represents an *MS1096-Gal4, UAS-Gαo*; *OK10-Gal4/UAS-Gαs[GTP]* fly.

Expression of the GTPase-deficient point mutant of Gαs induces precocious cell death, which results in hemolymph accumulation between the two epithelial wing sheets and wing blistering [22], [23], Fig. 6A. In mammalian systems Gαs can be overactivated by cholera toxin, which covalently ADP-ribosylates a conserved arginine residue of the GTPase active center [32]. To test whether cholera toxin was also active against *Drosophila* Gαs, we expressed the toxin in developing *Drosophila* wings, and found wing blistering induced by the toxin (Fig. 6B) similar to that induced by the constitutively activated Gαs (Fig. 6A). These data not only extend the known similarity between mammalian and fly Gαs, but they also demonstrate that targeted activation of the endogenous, not overexpressed, Gαs is sufficient to overactivate the pathway and produce wing blistering.

Cholera toxin-mediated activation of Gαs mimics that achieved by GPCR-mediated activation and results in production of GTP-loaded Gαs and free Gβγ subunits. As the latter can induce signal transduction in some instances [9], we investigated the effects of direct co-overexpression of Gβ13F/Gγ1 in *Drosophila* wings using a number of *Gal4* drivers lines. Gβ13F or Gγ1 subunits expressed alone were ineffective in inducing phenotypes (Fig. 6C, D). Despite the fact that co-overexpression of Gβ13F and Gγ1 could affect asymmetric cell divisions [17], Wnt/Frizzled signaling [14], planar cell polarity (data not shown), and venation (Fig. 6E), Gβ13F/Gγ1 was in no condition able to mimic the wing blistering phenotype induced by activation of Gαs (Fig. 6E). We also boosted Gβ13F/Gγ1 overexpression by combining two copies of the *UAS-Gβ13F, UAS-Gγ1* transgenes, as well as by providing two copies of the Gal4 driver lines; these attempts also failed to produce the wing blistering phenotype. These results demonstrate that the Gβγ heterodimer is required for the proper Gαs signaling, but by itself plays only the passive, permissive role in the signal transduction leading to apoptosis.

To further prove that Gβγ is not necessary for the execution of the apoptosis program once the activated Gαs is released, we co-expressed Gαs[GTP] with the wild-type Gαo sequestering the Gβγ subunits. We found that the potency of Gαs[GTP] to induce wing blistering was not at all affected by such Gβγ sequestration (Fig. 6F).

However, Gβγ might have a separate function in the Gs signaling, namely the induction of the epithelial-mesenchymal transition sub-pathway. Indeed, while co-overexpression of Gαs is capable of rescuing the folded wing phenotype induced by overexpression of Gαo, the constitutively activated form of Gαs is much less potent in performing such a rescue (Table 2). These data suggest that it is not the GTP-loaded Gαs, but the free Gβγ heterodimer, released from the heterotrimeric Gs complex upon *rickets* or other GPCR receptor activation, which is required for the epithelial-mesenchymal transition and wing expansion. This issue is further discussed in the next section.

## Discussion

The soft folded wings of the young insect freshly hatched from the pupal case within 1–2 hours expand and harden, becoming a robust flight organ. This process is accompanied by epithelial-mesenchymal transition and cell death of the wing epithelial cells [20], [21]. Genetic dissection has revealed the function of the neurohormone bursicon and its wing epithelial receptor *rickets* in initiation of these processes [21], [22], [24]. The GPCR *rickets* couples to the heterotrimeric G protein Gs; the Gαs-activated cAMP-PKA pathway culminates at the induction of apoptosis [22]. However, the overall phenotypic consequences of the loss of the Gs signaling pathway in post-eclosion wings were unknown, as well as the nature of the Gβγ subunits of the heterotrimeric Gs complex responding to the bursicon-*rickets* signaling.

Here we have performed an extensive analysis of the heterotrimeric G protein subunits in these post-eclosion stages of wing maturation. We find that the whole-wing down-regulation of Gαs results in the failure of wing expansion, demonstrating that this change in the shape of the wing is the major morphological outcome of the bursicon-*rickets*-Gs signaling. We also identify the Gβ13F and Gγ1 subunits as the other two constituents of the heterotrimeric Gs complex, as downregulation of Gαs, Gβ13F, or Gγ1, but not any other Gα, Gβ, or Gγ subunits encoded by the *Drosophila* genome, each leads to the same folded wing phenotype.

We also show that Gαo, but not any other Gα-subunit, can inhibit the wing expansion program through sequestration of the Gβ13F/Gγ1 heterodimer. The reason for the specificity of Gαo over other Gα-subunits in antagonizing the Gs signaling is unclear. It is unlikely that differences in expression levels of the tested Gα-subunits may account for the selective activity of Gαo. Indeed, most overexpression experiments were done with the X-chromosome-inserted *MS1096-Gal4* driver, which results in markedly higher expression levels in males than heterozygous female flies, producing a more penetrant folded wing phenotype in males overexpressing Gαo (see Table 2). However, even in male flies overexpressing other Gα-subunits no instances of the folded wing phenotype could be seen. Furthermore, several independent insertions of the UAS-Gα transgenes were tested; while different Gαo transgenes all produced the folded wing phenotype upon overexpression, other Gα constructs remained ineffective (data not shown).

Similarly, the different Gα-subunits possess a similar affinity towards the interaction with the Gβγ heterodimer [33], [34], not providing an explanation for a specific ability of Gαo to antagonize the Gs-mediated post-eclosion pathway. We are thus tempted to propose that a previously uncharacterized biochemical mechanism may allow for a specific antagonism physiologically existing between the Gs- and Go- mediated signaling pathways. As liberation of high amounts of GDP-loaded Gαo is predicted to be a consequence of activation of multiple Go-coupled GPCRs [33], and as Go is a heavily expressed G protein representing the major G protein species e.g. in the brain of flies and mammals [35], [36], this specific ability of Gαo to antagonize the Gs-mediated signaling may have physiological implications in other tissues and organisms than *Drosophila* wing. However, we would like to add that these speculations are based on the analysis of the overexpression data and must be treated with caution when translating them into physiological situations.

Only the GDP-loaded, but not the activated GTP-loaded form of Gαo is effective in antagonizing Gs. We have performed a proteomics analysis of the *Drosophila* proteins which would discriminate between the two nucleotide forms of Gαo, and revealed surprisingly few targets of this kind. While the chaperone Hsc70-3 and β1-tubulin preferentially interacted with the GTP-loaded Gαo, Gβ13F was found to specifically interact with Gαo-GDP. These data suggest that many Gαo-interaction partners do not discriminate between the two guanine forms of Gαo. These findings are in agreement with our other experimental findings [16], as well as our mathematical modeling predicting that high concentrations of free (monomeric) signaling-competent Gαo-GDP are produced upon activation of Go-coupled GPCRs [33].

Gαo-mediated sequestration of Gβ13F/Gγ1 depletes the pool of the heterotrimeric Gs complexes. As only heterotrimeric Gαβγ, but not monomeric Gα proteins can efficiently bind and be activated by their cognate GPCRs [4], [34], overexpression of Gαo abrogates the *rickets*-Gs signaling. Phenotypic consequences of this abrogation are the failures of apoptosis and wing expansion. In contrast, expression of the constitutively activated form of Gαs induces premature cell death and wing blistering [22], [23]. We find that this phenotype can be also induced by expression of cholera toxin, revealing that the ability of cholera toxin to specifically overactivate Gαs reported in mammalian systems [32] is reproduced with *Drosophila* proteins. These data also confirm that not only exogenously overexpressed, but also the endogenous Gαs can induce the precocious cell death upon overactivation.

**Figure 7:**
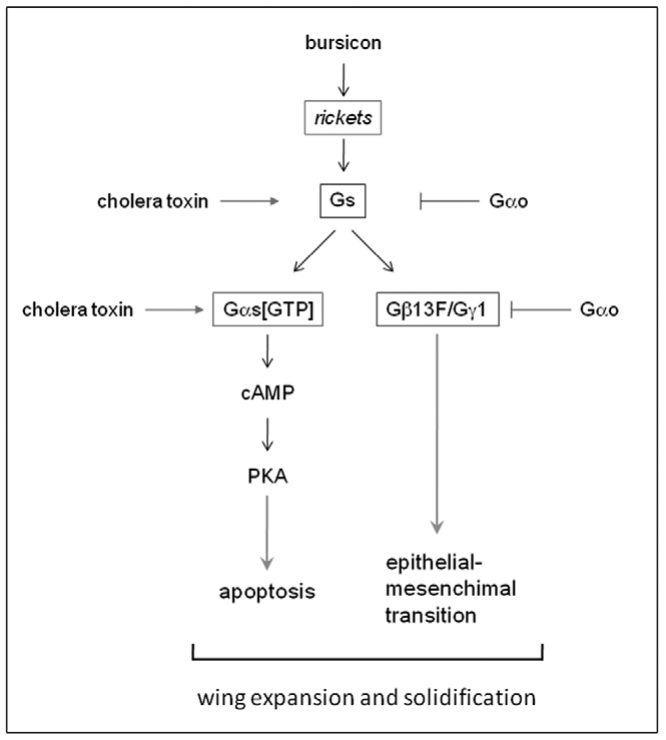
A model of the action of components of the heterotrimeric Gs complex in wing maturation. The neurohormone bursicon acts on the Gs-coupled GPCR *rickets* expressed in wing cells. The GPCR activity leads to dissociation of the heterotrimeric Gs complex into GTP-loaded Gαs and free Gβγ-heterodimer. Gαs[GTP] activates the cAMP-PKA pathway to promote apoptosis. Gβγ, on the other hand, acts to induce the epithelial-mesenchymal transition. These two processes, acting in coordination, lead to post-eclosion wing expansion and solidification. Expression of the constitutively active Gαs or cholera toxin stimulates the Gαs-dependent branch in this signaling. Expression of Gαo inhibits this signaling through sequestration of the Gβγ-subunits.

However, prevention of apoptosis is not sufficient to produce the folded wing phenotype (Fig. 5). Together with the observation that the constitutively active form of Gαs is ineffective in rescuing the wing expansion defects produced by Gαo overexpression (Table 2), these data suggest that the Gαs-cAMP-PKA pathway culminating at apoptosis is not the sole signaling branch emanating from the bursicon-*rickets* GPCR activation. We propose that the second signaling branch initiated by the *rickets*-mediated dissociation of the heterotrimeric Gs complex is represented by the free Gβγ subunits, signaling to epithelial-mesenchymal transition (Fig. 7). Such a double signaling impact mediated by the two components of the heterotrimeric G protein complex leads to initiation of two cellular programs - apoptosis and epithelial-mesenchymal transition - which cumulatively result in wing expansion and solidification (Fig. 7), producing the adult flight organ. This two-fold response of the *Drosophila* wing to the maturation signal, mediated by the two components of the heterotrimeric G protein complex activated by the single hormone-responsive GPCR, provides an elegant paradigm for the coordination of signaling and developmental programs.

## Materials and Methods

### Fly stocks and crosses

The following *Drosophila* lines were used: *MS1096-Gal4* [25] and *OK10-Gal4* [23]; *Vg-Gal4, UAS-flp* [37]; *UAS-Gαo* and *UAS-Gαo[GTP]* [15]; *UAS-Gαi* [7]; *UAS-Gαs* and *UAS-Gαs[GTP]* [23]; *UAS-Gαq* [38]; *UAS-Gβ13F* [17]; *UAS-Gγ1* [8]; *cta^1^* [13]; *UAS-p35* [39]; and *UAS-GFP* and *Gβ76C^1^* (Bloomington Stock Center). The *UAS-RNAi* lines were from VDRC [40]; *UAS-GαqRNAi* was additionally from [31]. All crosses were performed at 25°C. The *UAS>w^+^>cholera toxin* [15] stock cannot be maintained without the flip-out cassette and thus must be crossed with a flipase-expressing line for Gal4-mediated expression of the toxin.

### Histology

All wings were prepared from ≥1 day-old flies and mounted in GMM as described [15]. For GFP, as well as for rhodamine phalloidin (Molecular Probes) visualization after treatment as described for imaginal discs [15], wings were mounted in Moviol. Whole young flies (≤1 day-old) were photographed through a Zeiss Stemi 2000 binocular using the Canon PowerShot G10 camera to visualize wing blistering. DAPI staining was performed after [21] with the following modifications: after fixation in 4% formaldehyde, the wings were successively treated at 17°C with chloroform for 1 h, heptane for 2 h, and 1× PBS/0.2% Tween 20 for 3 h, prior to the overnight DAPI (1∶10000, Sigma) staining at 17°C. These modifications aimed at increasing accessibility of DAPI to the folded aged wings.

### Recombinant Gαo expression

(His)_6_-tagged Gαo expression in *E.coli*, purification, immobilization on CNBr (cyanogen bromide)-activated sepharose, and specific activity measurements were performed as described [16].

### Proteomic analysis of Gαo-interacting partners

*Drosophila* head extracts were applied to the CNBr-immobilized Gαo preloaded with GDP or GTPγS as described [16]. The incubation slurry was packed into a 1 ml polypropylene column (Qiagene) and washed 3 times with 10 bed volumes of the binding buffer (50 mM Hepes pH 7.5, 100 mM KCl, 10 mM NaCl, 5% glycerol, 2 mM EGTA, 1× complete EDTA-free protease inhibitor cocktail (Roche), 0.5% Nonidet P-40, 0.1% Tween20) at 4°C. Retained proteins were eluted by 8 M Urea, separated by SDS-PAGE, and silver-stained. Alternatively, the eluted proteins were precipitated by methanol/chloroform [41] for mass spectrometry analysis.

Fifty µg of the precipitated proteins were labeled with CyDye DIGE Fluor minimal dyes according to the manufacturer recommendations (GE Healthcare Life Sciences). The samples were cup-loaded onto 24 cm pH 3–11 IEF strips and electrofocused with a total of 45′000 Vh using an Ettan IPGphor II (both GE Healthcare Live Sciences). The strips were reduced and alkylated according to the manufacturer recommendations. The second dimension separation was performed on 10–15% linear gradient gels automatically casted using a2DEoptimizer (NextGen Sciences) and the gels were run in the Ettan Dalt II (GE Healthcare Live Sciences) at 25°C. The gels were scanned using a Typhoon 9200 scanner (GE Healthcare Life Sciences). The gel images were analyzed using SameSpots (Nonlinear Dynamics) involving automatic normalization and automatic background substraction.

After subsequent Coomassie staining, spots of interest were picked using GelPal (Genetix) and digested overnight at 37°C (19 ng trypsin (Promega) in 47 mM Tris pH 9.0). The peptides were analyzed using LC-MSMS (4000 Q TRAP, Applied Biosystems) and proteins were identified using Mascot (Matrix Science) searching the protein sequence database UNIPROT-15.6.
